# Mediation of the relationship between cerebral blood flow and functional outcomes by quantification of perforator collateral capacity in patients with minor stroke and large vessel occlusion

**DOI:** 10.3389/fneur.2026.1915617

**Published:** 2026-07-30

**Authors:** Jing Lei, Sifei Wang, Man Gao, Yan Ding, Yu Guo

**Affiliations:** 1 Department of Radiology, Tianjin Huanhu Hospital, Tianjin, China; 2 Department of Neurosurgery, Tianjin Huanhu Hospital, Tianjin, China; 3 Medical School, Tianjin University, Tianjin, China

**Keywords:** acute ischemic stroke, cerebral blood flow, endovascular therapy, high-resolution magnetic resonance imaging, lenticulostriate artery

## Abstract

**Purpose:**

Recognition of lenticulostriate arteries (LSAs) may reflect the presence of beneficial local collaterals. This study determined whether the morphological characteristics of LSAs were associated with clinical outcomes in individuals with intracranial atherosclerosis–related large vessel occlusion (ICAS-LVO) and minor stroke who underwent endovascular treatment and examined the potential hemodynamic relevance of these features.

**Materials and methods:**

This retrospective observational study included consecutive individuals with ICAS-LVO and minor stroke who underwent successful endovascular treatment between June 2020 and March 2022. LSA morphology was assessed on preinterventional high-resolution magnetic resonance imaging (HR-MRI) and classified as relatively good (LSA-sign+) or reduced (LSA-sign−). Cerebral blood flow in the LSA territory was quantified using arterial spin labeling. Associations between LSA characteristics, cerebral blood flow, and clinical outcomes were evaluated using multivariable logistic regression and Spearman correlation analyses, adjusted for potential confounders.

**Results:**

Of 52 individuals included (median age, 59 years; 40 men), 24 (46.2%) had LSA-sign+. Multivariable analysis revealed that longer total LSA length was independently associated with higher odds of favorable outcome at 90 days (modified Rankin Scale score ≤2; OR = 1.14; 95% CI, 1.02–1.27; *p* = 0.02). Model performance improved with inclusion of the LSA feature. Mediation analysis demonstrated that the association between LSA characteristics and favorable outcome was mediated by cerebral blood flow (indirect effect = 0.16; 95% CI, 0.03–21.40).

**Conclusion:**

Total LSA length on preinterventional HR-MRI was an independent predictor of favorable outcomes after endovascular treatment. These findings provide new insight that may support clinical decision-making in ICAS-LVO and minor stroke.

## Introduction

Presence of large vessel occlusion (LVO) in individuals with minor stroke is associated with a high risk of adverse outcomes (1). Several studies have examined the benefits of immediate endovascular treatment in this population (2–4). However, the substantial risk of poor 3-month functional outcomes has been consistently reported. For individuals who are likely to have a poor prognosis, optimal medical management when appropriate, followed by rescue mechanical thrombectomy in the event of clinical deterioration, may be more suitable. Thus, patient selection and outcome prediction remain major concerns for endovascular treatment in minor stroke. Deep brain tissue has limited collateral circulation, which may contribute to futile recanalization (5, 6). Regions such as the internal capsule heavily depend on lenticulostriate arteries (LSAs) for blood supply, and these arteries support key motor and sensory fiber pathways. When LSAs are obstructed, irreversible ischemic injury can occur, and even rapid and successful revascularization may not reverse the resulting deficits.

Previous studies reporting these findings have been primarily conducted in Western populations without intracranial atherosclerosis (ICAS) as the underlying stroke mechanism, limiting generalizability to populations with a higher burden of anterior-circulation atherosclerotic disease. Asian populations have a markedly higher prevalence of ICAS than Western populations (7). In addition, a previous study identified local collateral networks that bridge the site of ICAS-related stenosis or occlusion (8). These collaterals appear as arterial networks connecting the proximal trunks of LSAs to distal vessels through parallel channels. Thus, LSAs supported by collateral flow may be present in individuals with ICAS-related LVO. However, whether these collateralized LSAs affect vasodynamics or clinical outcomes remains uncertain.

Historically, cerebral collaterals were viewed merely as static anatomical vascular structures. Modern research (9, 10) has redefined collateral circulation as a key physiological factor governing infarct progression, tissue viability, reperfusion benefit, and long-term functional recovery following LVO. Collateral perfusion capacity exerts a stronger influence on penumbral salvage and infarct expansion speed than fixed time cutoffs, and it informs personalized reperfusion treatment strategies. Perforator collateral perfusion capacity refers to the maximum perfusion compensation potential of native intracranial penetrating arterial anastomoses after vessel occlusion, a property largely determined by the inherent anatomical morphology of LSAs (11, 12). Although collateral circulation functions as a dynamic physiological process modulated by post-occlusion variables such as cerebral autoregulation, microvascular integrity, and time-dependent vascular remodeling, LSA anatomy still serves as a steady, primary marker of baseline deep perforator collateral reserve. Conventional collateral grading scales predominantly assess superficial leptomeningeal collaterals, whereas deep striatocapsular perforator networks reflected by LSAs remain insufficiently characterized. This unmet research demand motivates the current study.

Digital subtraction angiography is the reference standard for assessing collateral circulation, but its invasive nature and its reliance on iodinated contrast and radiation limit widespread use. A recent study (13) reported that LSAs can be evaluated noninvasively by using time-of-flight MR angiography (TOF-MRA). However, LSAs are often not visible on conventional MRA, and 7T-MRA, which provides superior spatial resolution, is not feasible for routine clinical practice. Several studies (14, 15) have demonstrated that high-resolution magnetic resonance imaging (HR-MRI) can directly visualize LSAs and exhibits good agreement with 7T-MRA (16). Quantitative cerebral blood flow can also be measured noninvasively by using arterial spin labeling (ASL) (17). Advances in ASL techniques, such as multi-delay labeling, pseudo-continuous labeling, and background suppression, have further improved measurement accuracy in deep brain structures (18). These developments provide an opportunity to examine whether collateral-supported LSAs are present in individuals with ICAS-LVO and whether these vessels affect cerebral blood flow and clinical outcomes.

We hypothesized that collateral-supported LSAs and corresponding cerebral blood flow can be identified using HR-MRI and ASL. Accordingly, in this study, we examined LSA morphology before recanalization, determined its association with clinical outcomes, and explored the underlying pathophysiologic mechanisms.

## Materials and methods

### Study patients

**Figure 1:**
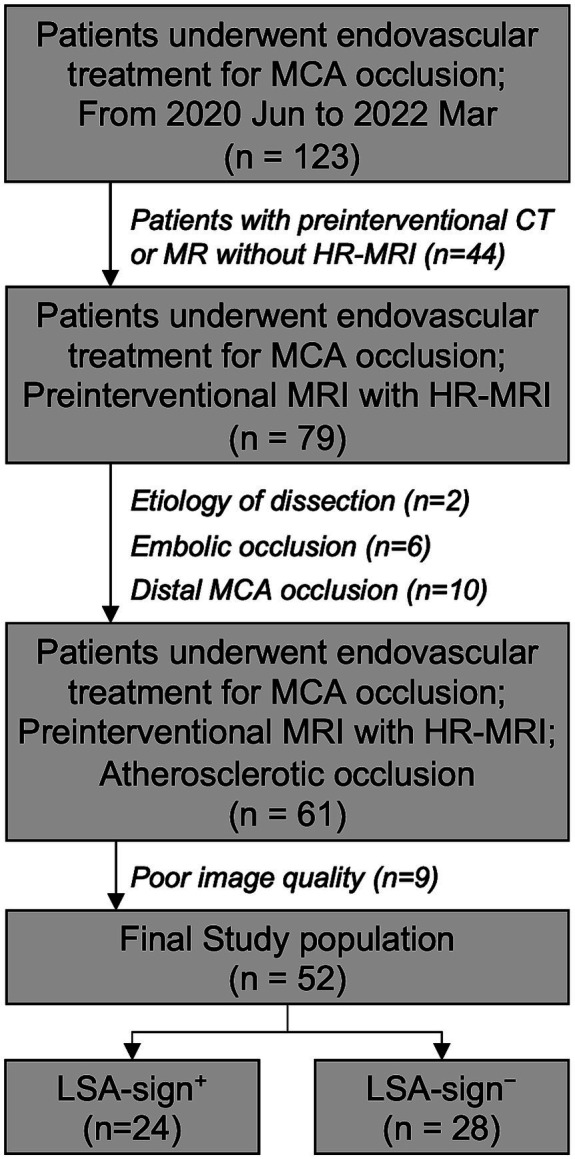
Flow chart of the patient enrollment.

This study was approved by the institutional ethics committee and conducted in accordance with the Declaration of Helsinki. Written informed consent was obtained from each participant. This retrospective observational study, derived from a prospective stroke registry (ClinicalTrials.gov: NCT0565916), included consecutive individuals with ICAS-LVO and mild symptoms who underwent successful endovascular treatment between June 2020 and March 2022. ICAS-LVO was defined as a significant fixed focal stenosis at the occlusion site, identified either on the final angiographic evaluation or during the endovascular procedure (19). The inclusion criteria were (1) a baseline National Institutes of Health Stroke Scale (NIHSS) score of 6 or less (20), (2) a proximal middle cerebral artery (MCA) occlusion involving the LSAs as detected on TOF-MRA, (3) successful recanalization after immediate endovascular treatment, and (4) availability of preinterventional MRI, including HR-MRI and ASL. Exclusion criteria were (1) non-diagnostic imaging due to motion artifacts, (2) greater than 50% stenosis of contralateral anterior circulation large vessels, and (3) non-atherosclerotic intracranial arterial disorders such as vasculitis, dissection, or iatrogenic occlusion. Hemispheres without a history of stroke were used as controls (21). The study flowchart is presented in Figure 1.

### Magnetic resonance imaging data acquisition

The MRI was performed on a 3T scanner (MAGNETOM Prisma, Siemens Healthcare, Erlangen, Germany) by using a 64-channel head–neck coil. HR-MRI was performed using a precontrast three-dimensional turbo spin-echo technique known as T1-weighted sampling perfection with application-optimized contrast using different flip angle evolutions. The HR-MRI parameters were as follows: repetition time (TR), 900 ms; echo time (TE), 15 ms; field of view, 200 × 200 mm^2^; matrix, 320 × 320; 224 slices; slice thickness, 0.53 mm; and acquisition time, 7 min. ASL imaging was performed using a five-delay pseudo-continuous ASL sequence with background-suppressed three-dimensional gradient-and-spin-echo readout. ASL parameters were as follows: TR, 4600 ms; TE, 30 ms; field of view, 22 × 22 mm^2^; matrix, 64 × 64; 32 slices; slice thickness, 3.5 mm; labeling duration, 1.5 s; and post-label delays of 1, 1.5, 2, 2.5, and 3 s. The acquisition time was 6 min. Additional sequences included diffusion-weighted imaging (DWI; TR, 2,900 ms; TE, 73 ms; *b*-value, 1,000 s/mm^2^; slice thickness, 5.0 mm; distance factor, 30%; acquisition time, 23 s) and TOF-MRA (TR, 21 ms; TE, 3.4 ms; slice thickness, 0.8 mm; distance factor, −32.5%; acquisition time, 3 min 44 s).

### Imaging analysis

#### LSA morphometry

Image analysis was performed using Syngo. Via (Siemens Healthcare, Erlangen, Germany). HR-MRI images were reconstructed as coronal minimum intensity projections with a thickness of 30 mm. For each participant, the number and total length of visualized LSA branches on the symptomatic side were measured. Only LSA branches measuring 5 mm or more were included in the analysis. When an artery branched within 5 mm of the MCA origin, each branch was counted and measured separately (22). When branching occurred more distally, only the longest branch was counted and measured.

#### LSA classification and LSA-sign

Visibility of the LSAs was graded on coronal 30-mm minimum intensity projection images from the HR-MRI by using the following scale (23): (0) faint linear visualization, (1) significantly reduced LSAs, (2) relatively normal LSA morphology, and (3) enlarged vessel diameter comparable to that of the contralateral LSAs.

#### Cerebral blood flow analysis

Postprocessing and analysis of ASL data were performed using Cereflow, a Java-based software package (Translational MRI, LLC, LA, CA). Cerebral blood flow maps were generated by averaging the estimated cerebral blood flow across all post-label delay times. After computation, a predefined template of the perforating MCA territory was applied to extract the mean cerebral blood flow values within the LSA territory.

### Endovascular treatment

All procedures followed local standard operating protocols and institutional guidelines. Treatment decisions and imaging evaluations were made by the physician responsible for diagnosis and management. At our center, MRI is the first-line imaging modality for candidates for reperfusion therapy. HR-MRI and perfusion imaging were used to assess procedural feasibility. Because operating room availability occasionally required brief adjustments in workflow, these imaging examinations did not delay treatment initiation. During reperfusion therapy, selective angiography was performed to determine the extent of the disease. After angiographic assessment, the treating physician selected the appropriate endovascular strategy. Successful recanalization was defined as a modified Thrombolysis in Cerebral Infarction grade of 2b or 3.

### Clinical and imaging variables

Clinical data collected for each participant included age, sex, admission NIHSS score, vascular risk factors, and time from symptom onset to arterial puncture. DWI lesion extent was assessed using the Alberta Stroke Program Early CT Score (DWI-ASPECTS). The presence or absence of symptomatic intracranial hemorrhage (sICH) was evaluated on postinterventional MR or CT images. sICH was defined as any intracranial hemorrhage associated with a ≥ 4-point increase in the NIHSS score within 24 h (24). Favorable outcome was defined as a modified Rankin Scale (mRS) score of ≤2 at 90 days.

### Statistical analysis

All analyses were performed using MedCalc software (version 11.1.1.0; Mariakerke, Belgium). A *p*-value of <0.05 was considered statistically significant. Continuous and ordinal variables are presented as median (interquartile range [IQR]), and categorical variables are presented as *n*/*N* (%). Independent group comparisons were conducted using the Mann–Whitney U test. Univariate logistic regression was performed to identify variables associated with clinical outcome, and variables with *p* < 0.1 were entered into the multivariate logistic regression model. Receiver operating characteristic curves and corresponding area under the curve values were generated to assess model performance. Spearman correlation analysis was performed to examine the association between LSA features and cerebral blood flow in the LSA territory. Mediation analysis was conducted using the PROCESS macro to further characterize the relationships among LSA features, cerebral blood flow, and favorable outcome. Three pathways were tested: (1) the association between LSA features and favorable outcome; (2) the association between LSA features and cerebral blood flow; and (3) the association between cerebral blood flow and favorable outcome. If all three pathways were significant, mediation (indirect effect, ab) was assessed by estimating the direct effect (pathway c′). Significance was evaluated using 5,000 bootstrap iterations, with mediation considered significant when the 95% bootstrap confidence interval (CI) did not include zero.

## Results

### Participants

Among 123 patients who underwent successful endovascular treatment for a proximal MCA occlusion, 52 patients (median age, 58.5 years; IQR, 52.0–67.5 years; male patients, 76.9%) were included. Patients with incomplete images (*n* = 44), distal MCA occlusion (*n* = 10), arterial dissection (*n* = 2), embolic occlusion (*n* = 6), and poor image quality (*n* = 9) were excluded. Because all patients in this study required imaging screening, the time to transfer patients to MRI before operation was justified. The final methods for achieving recanalization included intracranial stenting in 57.7% (*n* = 30), balloon angioplasty in 28.8% (*n* = 15), intra-arterial tirofiban infusion in 5.8% (*n* = 3), and mechanical thrombectomy alone in 7.7% (*n* = 4). The treatment methods were balanced between the groups. On HR-MRI, 24 patients (46.2%) had good LSAs (LSA-sign+; grade 2, *n* = 18; grade 3, *n* = 6) and 28 patients (53.8%) had reduced LSAs (LSA-sign−; grade 0, *n* = 6; grade 1, *n* = 22). Detail information of clinical and image characteristics are summarized in Table 1. No significant differences were observed between the two groups in terms of clinical data, time since onset to puncture, occlusion length, and DWI-ASPECTS.

**Table 1:** Characteristics with LSA-sign^+^ versus LSA-sign^−^.

Characteristics	LSA-sign^+^ (*n* = 24)	LSA-sign^−^ (*n* = 28)	*p*-value
Age, years	58.5 (52.0 ~ 67.5)	58.5 (51.5 ~ 65.5)	0.72
Male, *n* (%)	18 (75.0%)	22 (78.6%)	0.77
Vascular risk factors, *n* (%)			
Hypertension	18 (75%)	20 (71.4%)	0.78
Diabetes mellitus	8 (33.3%)	3 (10.7%)	0.06
Hyperlipidemia	0 (0%)	2 (7.1%)	0.28
Atrial fibrillation	0 (0%)	1 (3.6%)	0.51
Admission NIHSS	4.0 (4.0 ~ 5.0)	5.0 (4.0 ~ 5.0)	0.99
DWI-ASPECTS	9.0 (8.0 ~ 10.0)	8.5 (7.0 ~ 9.5)	0.23
Occlusion length, mm	14.0 (10.8 ~ 16.8)	13.8 (11.7 ~ 15.7)	0.99
Onset-to-puncture, h	11.5 (10.0 ~ 13.4)	11.5 (10.1 ~ 14.1)	0.71
Imaging-to puncture, h	1 (0.8 ~ 1.2)	1 (0.9 ~ 1.2)	0.6
Procedural details			0.21
MT alone	3 (12.5%)	1 (3.6%)	
ITI + MT	2 (8.3%)	1 (3.6%)	
BA + ITI + MT	7 (29.2%)	8 (28.6%)	
Stenting + BA + ITI + MT	12 (50%)	18 (64.3%)	
Morphological characteristics of the LSAs			
Branch number	3 (3 ~ 3)	3 (2 ~ 3)	0.06
Total length, mm	38.6 (35.6 ~ 44.7)	23.8 (18.8 ~ 27.2)	<0.001^*^
Cerebral blood flow, ml/100 g/min			
LSA territory	41.6 (33.3 ~ 49.5)	26.5 (21.2 ~ 30.5)	<0.001^*^
Clinical outcomes, *n* (%)			
Symptomatic ICH	1 (4.2%)	1 (3.6%)	0.93
Favorable outcome	21 (87.5%)	16 (57.1%)	0.02^*^

Data are presented as *n* (%) or as median (interquartile range). BA, balloon angioplasty. DWI-ASPECTS, diffusion-weighted imaging lesion extent using the Alberta Stroke Program Early CT Score. ICH, intracerebral hemorrhage. ITI, intra-arterial tirofiban infusion. LSA, lenticulostriate artery. MT, mechanical thrombectomy. NIHSS, National Institutes of Health Stroke Scale. ^*^p < 0.05.

### LSA distribution and perforator cerebral blood flow

**Figure 2:**
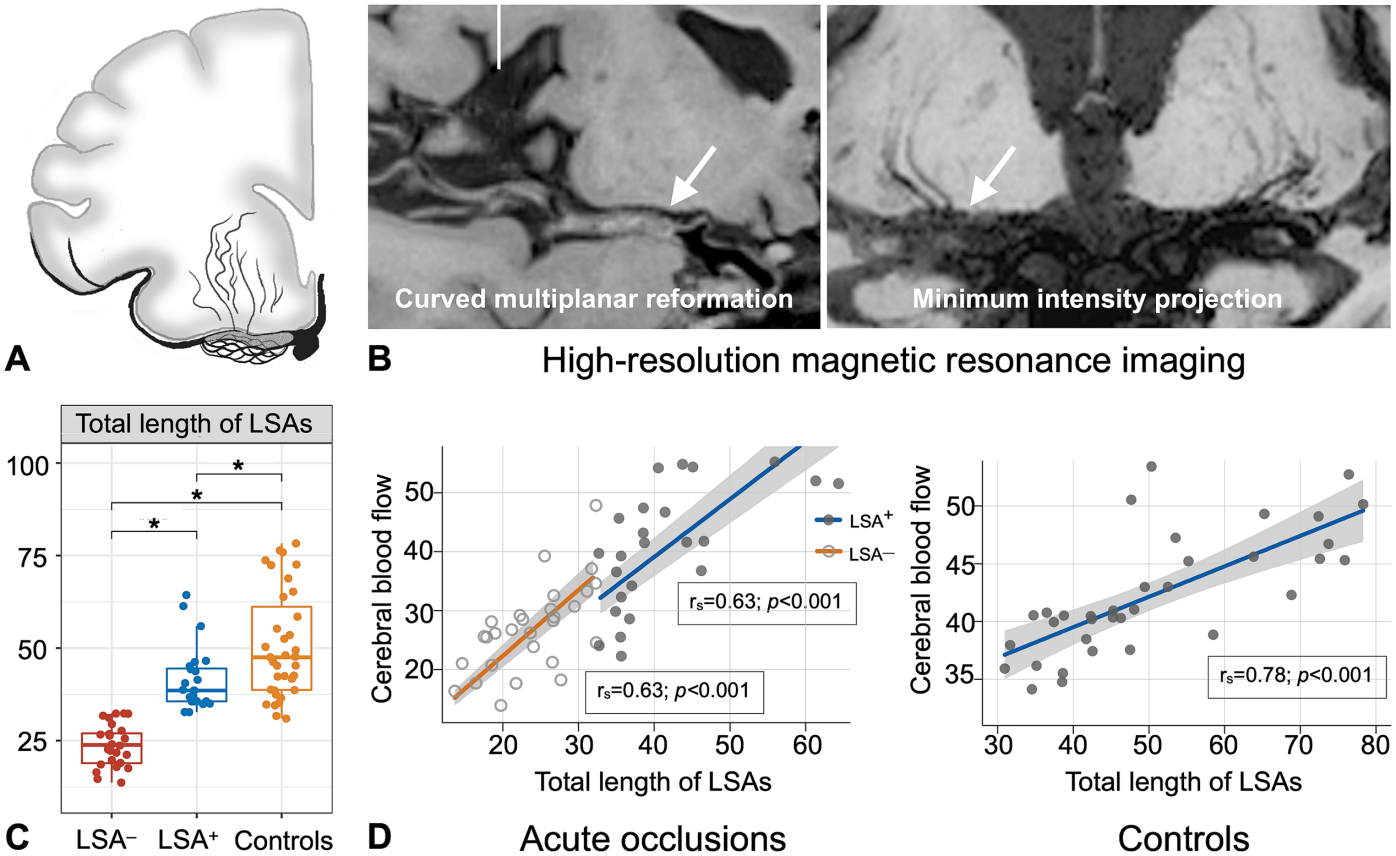
Diagram **(A)** and corresponding high-resolution magnetic resonance imaging images **(B)** of lenticulostriate arteries (LSA) compensated by local collateral networks bridging the site of occlusion. **(C)** Plots comparing the total length of lenticulostriate arteries (LSA). Box-and-whisker plots represent medians, interquartile ranges, and greatest and least values. ^*^Statistically significant, *p* < 0.05. **(D)** Scatter plots of the correlations between the total length of LSAs and cerebral blood flow in LSA territory.

A total of 87 hemispheres were analyzed, including 24 in the LSA-sign+ group, 28 in the LSA-sign− group, and 35 controls. In the LSA-sign+ group, all patients exhibited irregular net-like vessels surrounding the occluded arterial segments. The number of LSA branches did not differ significantly between the LSA-sign+ and LSA-sign− groups (*p* = 0.06). However, the median total length of visualized LSA branches differed significantly among the three groups based on the Kruskal–Wallis test (*p* < 0.001). The LSA-sign− group had a significantly shorter total length of LSA branches (23.8 mm [IQR, 18.8–27.2]) compared with both the LSA-sign+ group (38.6 mm [IQR, 35.6–44.7]; *p* < 0.001) and controls (47.5 mm [IQR, 38.7–62.5]; *p* < 0.001; Figures 2A–C).

Median cerebral blood flow within the LSA territory was 26.5 mL/100 g/min (IQR, 21.2–30.5) in the LSA-sign− group, 41.6 mL/100 g/min (IQR, 33.3–49.5) in the LSA-sign+ group, and 47.8 mL/100 g/min (IQR, 38.6–45.6) in controls. The total length of visualized LSA branches were significantly and positively correlated with cerebral blood flow in both acute occlusions and controls (all rs > 0.60; *p* < 0.001; Figure 2D).

### Clinical outcomes

**Figure 3:**
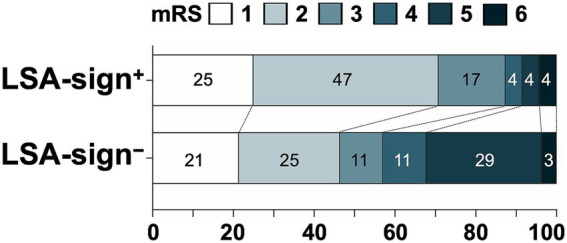
Distribution of scores on the Modified Rankin Scale (mRS) at 90 days. Shown is the distribution of scores for disability on the mRS (which ranges from 0 to 6, with higher scores indicating more severe disability) among patients in the lenticulostriate artery (LSA)-sign^+^ group and the LSA-sign^−^ group. The numbers in the bars are the percentages of patients who had each score.

The sICH occurred in 4.2% of individuals with LSA-sign+, which was not significantly different from the rate in those with LSA-sign− (3.6%; *p* = 0.93). Individuals with LSA-sign+ had a markedly higher rate of favorable outcome compared with those with LSA-sign− (87.5% vs. 57.1%; *p* = 0.02; Figure 3).

**Figure 4:**
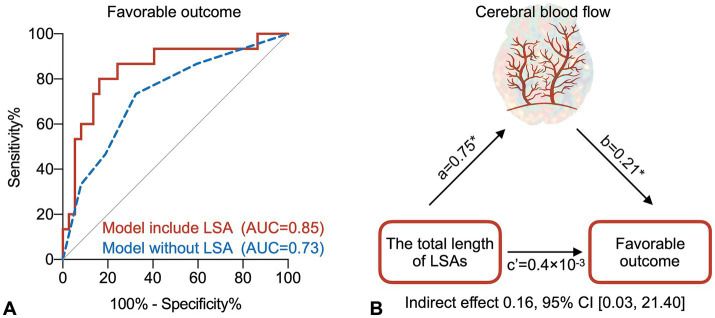
**(A)** Prediction improvement in ROC analysis. The model including the lenticulostriate artery (LSA) feature (red) had superior AUC as compared to the model without the LSA feature (blue). **(B)** The mediation analyses between the LSA feature (a) and favorable outcome (b), with cerebral blood flow in LSA territory as the mediator (*c’*). ^*^Statistically significant, *p* < 0.05.

After adjustment for baseline variables with *p* < 0.1 in univariable analyses, the total length of visualized LSA branches was independently associated with favorable outcome (odds ratio [OR], 1.13; 95% CI, 1.03–1.23; *p* = 0.01). DWI-ASPECTS was also an independent predictor in the multivariate logistic regression model (OR, 1.72; 95% CI, 1.03–2.86; *p* = 0.04). Detailed results are presented in Table 2. Model performance improved when the LSA feature was incorporated, as reflected by a higher area under the curve for prediction of favorable outcomes (0.85 vs. 0.73; Figure 4A). In the mediation analysis, all three prerequisite associations were confirmed. The final step demonstrated that cerebral blood flow in the LSA territory mediated the association between the LSA feature and favorable outcomes (indirect effect, 0.16; 95% CI, 0.03–21.40; Figure 4B).

**Table 2:** Predictors of favorable outcome based on multiple logistic regression analysis.

Characteristics	Univariate analysis			Multivariate analysis		
	OR	95% CI	*p*-value	OR	95% CI	*p*-value
Age	1.02	0.97 ~ 1.07	0.43	NA		
Admission NIHSS	1.25	0.67 ~ 2.34	0.48	NA		
Treatment methods	0.83	0.41 ~ 1.69	0.60	NA		
Time to treatment	0.87	0.69 ~ 1.10	0.25	NA		
DWI-ASPECTS	1.84	1.116 ~ 2.91	0.006^*^	1.72	1.03 ~ 2.86	0.04
Total length of LSAs	1.14	1.04 ~ 1.24	0.04^*^	1.13	1.03 ~ 1.23	0.01

Data are presented as *n* (%) or as median (interquartile range). CI, confidence interval. DWI-ASPECTS, diffusion-weighted imaging lesion extent using the Alberta Stroke Program Early CT Score. LSA, lenticulostriate artery. NA, not applicable. NIHSS, National Institutes of Health Stroke Scale. OR, odds ratio. ^*^p < 0.1.

### Reproducibility

Two reviewers (JL and MG, with 20 and 10 years of neuroradiology experience, respectively) independently analyzed the HR-MRI datasets. Both reviewers, each with at least 10 years of neuroradiology training, were blinded to all clinical information. To assess intraobserver reliability, one reviewer repeated the evaluation 14 days later while remaining blinded to the initial assessment. Inter- and intra-observer agreement was quantified using the intraclass correlation coefficient (ICC). Any discrepancies between the two reviewers were resolved by consensus. Intraobserver and interobserver agreement (ICC = 0.97; 95% CI, 0.95–0.98) for LSA grading were excellent (ICC = 0.98; 95% CI, 0.97–0.98). Similarly, intra-observer agreement for LSA morphometric measurements was high for both total length (ICC = 0.96; 95% CI, 0.93–0.98) and branch number (ICC = 0.98; 95% CI, 0.96–0.99). Inter-observer agreement was also excellent for total length (ICC = 0.91; 95% CI, 0.84–0.95) and branch number (ICC = 0.96; 95% CI, 0.93–0.98). Supplementary Figure 1 shows Bland–Altman plots for intra- and inter-observer agreement of LSA quantitative and grading metrics. Reproducibility was further assessed using the coefficient of variation (standard deviation divided by mean × 100%). The maximal coefficient of variation was 18.0% (range, 10.7–18.0%). Based on this value, a sample size of 44 would be required to detect a 15% difference with 80% power at a significance level of 0.05. Because the between-group differences in our study exceeded 15%, the sample size was sufficient to support robust conclusions.

## Discussion

This study used a high-resolution magnetic resonance imaging technique to achieve direct *in vivo* visualization of lenticulostriate arteries before endovascular treatment in individuals with atherosclerotic occlusion and minor stroke. To our knowledge, this is the first study to use imaging to characterize the relationship between lenticulostriate arterial morphology and cerebral blood flow, providing new insight into the vasodynamic mechanisms relevant to stroke. In addition to clinical variables, the total length of lenticulostriate arteries was independently associated with a higher likelihood of favorable 90-day outcomes (OR = 1.13; 95% CI, 1.03–1.23; *p* = 0.01). Incorporating this imaging marker may therefore enhance prediction of functional outcome.

Despite longstanding assumptions that the striatocapsular region is supplied by non-collateralized end arteries, collateral-supported LSAs in individuals with ICAS-LVO have been largely overlooked and rarely described. A previous study (8) using four-dimensional digital subtraction angiography demonstrated the presence of local collateral networks bridging the site of obstruction. Similar collateral patterns have been reported in moyamoya disease (25, 26). In that condition, moyamoya vessels arising from the LSA territory serve as critical collateral pathways (27). In our cohort, differences in LSA visibility grades on HR-MRI suggest variable collateral support in acute ICAS-LVO. Accurate identification of the anatomic basis and capacity of lenticulostriate collateralization may improve risk assessment in individuals undergoing endovascular treatment.

The LSAs are perforating arteries that supply the internal capsule and basal ganglia. The internal capsule is a white matter structure, and previous studies (5, 28) suggest that white matter has greater ischemic tolerance than gray matter. Differences in cellular composition between gray and white matter correspond to different baseline levels of cerebral blood flow, which contributes to their variable susceptibility to hypoperfusion. Evidence from mechanical thrombectomy studies also indicates that successful recanalization preferentially salvages white matter instead of gray matter within the MCA territory. Consistent with these findings, we observed that the total length of visualized LSA branches was directly proportional to cerebral blood flow within the LSA territory. Higher cerebral blood flow may help protect the internal capsule from ischemic injury, which may explain why individuals in the LSA-sign+ group exhibited a significantly higher rate of favorable outcome.

Well-developed collateral vessels can maintain viable ischemic penumbra for up to 48 h in select patients and slow infarct growth, resulting in highly variable patterns of lesion progression among individuals (29, 30). Traditional rigid time cutoffs for intravenous thrombolysis and endovascular thrombectomy originate from pivotal clinical trials but fail to account for broad interpatient differences in innate collateral perfusion. In a multicenter registry enrolling 805 patients with LVO, Leng et al. (31) reported comparable baseline collateral status between atherosclerotic and cardioembolic stroke, though collaterals conferred distinct clinical advantages across stroke subtypes: lower hemorrhagic complication rates in atherosclerotic stroke and improved long-term functional recovery in cardioembolic stroke. Separately, Salim et al. (32) established the collateral impairment score, a comprehensive CTA grading system incorporating both arterial and venous collateral grades. This combined metric independently predicted severe disability and higher 90-day mortality in patients with anterior circulation LVO, with risk rising alongside worsening collateral impairment. Consistent with these prior findings, our study demonstrated that patients with preserved LSA collateral networks (LSA-sign+) exhibited markedly higher cerebral blood flow within LSA territories, alongside a favorable 90-day functional outcome rate of 87.5%.

Collectively, accumulating clinical evidence argues against rigid, time-only therapeutic windows, positioning collateral circulation as the key factor governing ischemic tissue survival. While early reperfusion therapy remains essential, interindividual variability in collateral function creates highly personalized therapeutic windows. Even initially robust collateral perfusion gradually declines without timely recanalization; additionally, favorable collateral scores do not negate hemorrhagic transformation risk among patients with large infarct cores (33). Against this physiology-guided stroke management framework, LSA morphology serves as a stable anatomical marker of deep perforator collateral reserve, forming a clear mechanistic connection between vascular structure, collateral blood flow, and long-term functional prognosis.

Patient selection and prognosis prediction remain central challenges in determining suitability for recanalization. Clinical outcomes after endovascular treatment vary widely, and infarct volume alone is a limited predictor of functional recovery (34). Factors such as age, admission NIHSS score, and time from symptom onset to puncture continue to warrant careful evaluation. The territory supplied by the lateral LSAs may offer additional prognostic information. A previous study (35) demonstrated that visualization of LSAs on digital subtraction angiography before thrombectomy was associated with favorable outcomes. Our findings align with this observation but extend it by using HR-MRI to visualize LSAs, suggesting a role of HR-MRI in preinterventional imaging assessment. HR-MRI provides high spatial resolution and superior vessel–wall contrast, and previous studies have demonstrated that it reliably depicts LSAs and their morphological changes in individuals with single subcortical infarctions, atherosclerotic plaque, and moyamoya disease (15, 21, 36). Additional studies (23, 37) have also reported that the appearance of LSAs on TOF-MRA after thrombectomy correlates with favorable outcome. By contrast, lenticulostriate anastomoses have been associated with anterior hemorrhage in moyamoya disease (25, 38). In our cohort, however, the rate of sICH did not differ significantly between the LSA-sign+ and LSA-sign− groups.

This study has several limitations. First, the sample size was relatively small, largely because of the strict inclusion and exclusion criteria. Second, the retrospective design meant that rescue modalities for ICAS-LVO were not applied under a standardized protocol; however, all patients were recanalized according to the treating physicians’ best clinical judgment. Third, broader clinical deployment of HR-MRI will require further scan-time reduction enabled by AI-based super-resolution reconstruction. Fourth, although all occlusions were located in the proximal MCA, it was not always possible to confirm that portions of the LSAs were completely spared from involvement. Finally, longitudinal studies are necessary to further characterize the prognostic value of LSA morphometry and to validate its role in outcome prediction.

## Conclusion

The morphological characteristics of lenticulostriate arteries on preinterventional high-resolution MRI may help predict clinical outcomes after endovascular treatment in individuals with atherosclerotic occlusion and minor stroke. Evaluating these arterial features could enhance outcome prediction beyond currently recognized imaging markers. In this study, a longer total length of lenticulostriate arteries was associated with higher cerebral blood flow, a relationship that may indicate improved functional outcomes. These findings suggest that lenticulostriate artery morphology can inform patient selection for endovascular treatment.

## Data Availability

The data analyzed in this study is subject to the following licenses/restrictions: this retrospective study used data from a prospective stroke registry (NCT0565916) at Tianjin Huanhu Hospital. Ethical approval was granted by the local IRB (No. 2020-4), which waived the need for informed consent due to the anonymized nature of the data. The study complied with the Declaration of Helsinki and maintained strict patient privacy. Requests to access these datasets should be directed to JL, tyleijing@163.com.
