# Supplementary material for: Mediation of the relationship between cerebral blood flow and functional outcomes by quantification of perforator collateral capacity in patients with minor stroke and large vessel occlusion
Source: Front Neurol. 2026 Jul 30;17:1915617. doi: 10.3389/fneur.2026.1915617 (PMC13468522; doi:10.3389/fneur.2026.1915617)
Supplement: Supplementary file 1 [file Table_1.DOCX]

**SUPPLEMENTARY FIGURES**

**Supplementary Figure 1. Bland-Altman plots for intra- and inter-observer agreement of LSA grading, total length and branch number**
